# The *Salmonella* Typhi SPI-2 injectisome enigma

**DOI:** 10.1099/mic.0.001405

**Published:** 2023-10-20

**Authors:** Teresa L. M. Thurston, David W. Holden

**Affiliations:** ^1^​ Department of Infectious Disease, Centre for Bacterial Resistance Biology, Imperial College London, London, SW7 2AZ, UK

**Keywords:** *Salmonella *Typhi, SPI-2, type III secretion system, injectisome, replication, macrophages, effectors

## Abstract

The *Salmonella* pathogenicity island 2 (SPI-2)-encoded type III secretion system (injectisome) is assembled following uptake of bacteria into vacuoles in mammalian cells. The injectisome translocates virulence proteins (effectors) into infected cells. Numerous studies have established the requirement for a functional SPI-2 injectisome for growth of *Salmonella* Typhimurium in mouse macrophages, but the results of similar studies involving *Salmonella* Typhi and human-derived macrophages are not consistent. It is important to clarify the functions of the *S*. Typhi SPI-2 injectisome, not least because an inactivated SPI-2 injectisome forms the basis for live attenuated *S*. Typhi vaccines that have undergone extensive trials in humans. Intracellular expression of injectisome genes and effector delivery take longer in the *S*. Typhi/human macrophage model than for *S*. Typhimurium and we propose that this could explain the conflicting results. Furthermore, strains of both *S*. Typhimurium and *S*. Typhi contain intact genes for several ‘core’ effectors. In *S*. Typhimurium these cooperate to regulate the vacuole membrane and contribute to intracellular bacterial replication; similar functions are therefore likely in *S*. Typhi.

## Full-Text

Serovars of *Salmonella enterica* are intracellular bacterial pathogens that cause different diseases among many different mammalian hosts. The broad-host-range serovar Typhimurium usually causes self-limiting gastroenteritis in otherwise healthy humans but can spread to cause invasive non-typhoidal *Salmonella* disease among individuals with impaired immunity. Serovars Typhi and Paratyphi are the causes of the systemic disease typhoid fever. Infection of certain mouse strains with *Salmonella* Typhimurium leads to substantial bacterial colonization and replication in macrophages in the liver and spleen [1, 2]. Although this systemic illness recapitulates some of the features of typhoid fever and has been used for decades to model the human disease, there are important differences in the pathology [3, 4] and virulence factors of *Salmonella* Typhi and non-typhoidal serovars, including the Typhi-specific capsule [5] and its toxin [6].

A major virulence system of *S *. enterica is the *Salmonella* pathogenicity island 2 (SPI-2)-encoded type III secretion system (hereafter referred to as the SPI-2 injectisome), which is assembled following uptake of bacteria into *Salmonella *-containing vacuoles (SCVs) in mammalian cells. The injectisome comprises a needle-like structure on the surface of the bacterial cell and translocon proteins that are secreted through the needle then insert into the vacuole membrane to form a pore. This provides a conduit for the translocation of up to 30 different virulence proteins (effectors) into infected cells, including epithelial cells, macrophages, dendritic cells and other cell types. Almost all sequenced strains of serovars of *S. enterica* carry intact copies of the 28 genes involved in the assembly and function of the SPI-2 needle and translocon [7–9], as well as several of its associated effectors [10].

Various mutations that inactivate the SPI-2 injectisome (generating SPI-2 null mutant strains) have been used to assess its overall contribution to intracellular growth and virulence. Whereas numerous studies have established the requirement for a functional injectisome to enable intracellular growth of *S*. Typhimurium [11, 12], the results of similar studies involving *S*. Typhi are not consistent. Some have shown that it is required for replication in human epithelial cells [13] and in human-derived macrophages [14–16]. However, other work has led to the conclusion that it does not contribute to replication in such macrophages [13, 17, 18]. It is important to try to reconcile these differing conclusions and clarify the functions of the *S*. Typhi SPI-2 injectisome. If its functions differ substantially from those of *S*. Typhimurium, then we need to understand the differences and their underlying mechanisms. An inactivated SPI-2 injectisome forms the basis for live attenuated *S*. Typhi vaccines that have undergone extensive trials in humans [19–22]. Therefore, from a clinical perspective, it is essential to know the basis of attenuation. Further, if substantially different from the functions of the *S*. Typhimurium SPI-2 injectisome, this would highlight further distinctions between the mouse infection model with *S*. Typhimurium and typhoid fever in humans.

Experiments using SPI-2 null mutants to assess the contribution of the injectisome to intracellular survival and growth in host cells lines have relied mainly on counting the number of intracellular colony-forming units (c.f.u.) of bacteria recovered at different times post-uptake. Forest *et al*. [17], examined growth of wild-type and SPI-2 null mutant *S*. Typhi in human-derived THP1 macrophages and reported no differences between these strains. However, while numbers of wild-type and SPI-2 mutant c.f.u. recovered from infected macrophages were similar at various time points up to 24 h post-uptake, at 48, 72 and 96 h post-uptake, consistently fewer SPI-2 mutant c.f.u. were recovered [17]. The differences at individual time points were not significant [17], but perhaps a statistical difference would have been revealed had the data been analysed as a time series.

A second study by the same group [18], involved a genome-wide transposon mutant screen of *S*. Typhi with serial 24 h passages of mutants through THP-1 macrophages, followed by comparison of input and output ratios with a fourfold change threshold as cut off. This led to the identification of 130 attenuated mutants, including 3 carrying mutations in *ssaN*, *ssaP* and *ssaQ*, which were significantly underrepresented in the output pools (Table S3 [18]). In *S*. Typhimurium, these genes are all essential for SPI-2 injectisome function [23, 24]. However, the authors discounted a role of SPI-2, citing unpublished evidence for lack of attenuation for an individual *ssaP* mutant [18]. The authors also mentioned that input intensities from the SPI-2 genes were higher than the average and that their output values were similar to the average output intensity. However, since the conversion of raw data to ratios should account for such variations, it seems possible that the identification of *ssaN*, *ssaP* and *ssaQ* mutants is meaningful.

In a third study, Reuter *et al*. [13], used both a c.f.u. assay and flow cytometry with fluorescent bacteria to assess the contribution of the SPI-2 injectisome on growth of *S*. Typhi in human-derived epithelial cells and U937 macrophages. In epithelial cells, loss of injectisome function caused an approximately eightfold reduction (c.f.u.) and fourfold reduction (flow cytometry) in intracellular proliferation. However, very little growth of either wild-type or SPI-2 mutant *S*. Typhi occurred in U937 macrophages at 24 h post-uptake [13], making it difficult to assess the contribution of SPI-2 effectors to bacterial replication.

In contrast to the work described above, two papers have reported an effect of a SPI-2 injectisome null mutation on intracellular growth in U937 macrophages at 48 h post-uptake. Khan *et al*. [14] used a SPI-2 null *aroC* double-mutant strain and compared it with an *aroC* single mutant, both in the presence and absence of additional aromatic compounds (to complement the *aroC* mutation). They showed that in both cases, the SPI-2 null mutation reduced the growth of *S*. Typhi within these cells. In follow-up work, Stratford *et al*. [16] used the same strains and found that in two separate comparisons, the SPI-2 injectisome accounted for a difference in intracellular growth of at least fourfold over 48 h. More recently, Hamblin *et al*. [15] measured the replication of SPI-2 null mutants in primary human and a human-derived THP-1 macrophage cell line. While no differences were detected by c.f.u., a more sensitive method based on replication-associated dilution of a fluorescent protein revealed a replication defect of SPI-2 mutant between 12 and 24 h.

Other tests for the involvement of SPI-2 in *S*. Typhi virulence have exploited mice engrafted with human haematopoietic stem cells (hu-SRC-SCID). Unlike non-engrafted mice, these animals support the growth of *S*. Typhi and were used to screen transposon mutant libraries for *in vivo* growth defects [25]. While no mutants affected in SPI-2 function were recovered in the screen, an additional experiment compared bacterial growth in mice inoculated with a 1 : 1 ratio of wild-type and a SPI-2 mutant *Salmonella *, as well as a direct measurement of SPI-2-dependent growth in THP-1 macrophages. These tests also failed to show any attenuation for the SPI-2 mutant over a 24 h infection period [25]. However, in similar tests done by Hamblin *et al*. but involving a lower dose of mixed inoculum and a 5 day infection period, fewer SPI-2 mutants than wild-type bacteria were recovered from spleens (but not livers) of mice [15].

A different mouse model for *S*. Typhi infection is based on immunodeficient Rag2^−/−^γc^−/−^ mice engrafted with human foetal liver haematopoietic stem and progenitor cells [26]. These mice accumulate large numbers of *S*. Typhi, which can be detected in haematopoietic cells in the spleen and a more persistent infection is established, allowing analysis up to 4 weeks post-inoculation. Therefore, it would be very interesting to examine SPI-2-dependent infection dynamics in these mice.

What can explain the apparently conflicting results from these studies? The Reuter *et al*. paper [13] also included a temporal analysis of SPI-2 gene expression in U937 macrophages, which revealed that the proportion of *S*. Typhimurium bacteria expressing the SPI-2 *ssaG* promoter was over 90 % at 8 h post-uptake, while for *S*. Typhi the percentage rose steadily from approximately 50 % at 8 h to 77 % at 24 h post-uptake. Similarly, expression from *S*. Typhi of a fluorescent reporter, derived from a fusion to the promoter of the SPI-2 injectisome effector gene *sifA*, was delayed at 8 and 16 h post-uptake in epithelial cells compared to *S*. Typhimurium, but reached similar levels of induction by 24 h [13]. Therefore, it seems likely that expression of injectisome genes and effector delivery take longer in the *S*. Typhi/human macrophage model than for *S*. Typhimurium, and in keeping with the results of Khan *et al*. [14], and Stratford *et al*. [16], indicates that a longer duration of infection might have revealed SPI-2 dependent growth phenotypes.

It should also be kept in mind that when using approaches based on c.f.u. counts or flow cytometry of constitutively fluorescent bacteria, any difference in macrophage cytotoxicity caused by wild-type or mutant bacteria has the potential to yield an underestimation of the true level of intracellular bacterial replication for one of the strains. Whether SPI-2 injectisome effectors of *S*. Typhi induce cytotoxicity has not been investigated, but many *S*. Typhi strains encode the effector SseL [10], which has been shown to be required for a delayed cytotoxic effect in *S*. Typhimurium-infected mouse macrophages [27]. Therefore, it is possible that the methods used for measuring net bacterial growth in macrophages underestimated the growth of *S*. Typhi with a functional SPI-2 injectisome.

The SPI-2 injectisome and its effectors have been studied extensively in serovar Typhimurium, frequently in the context of growth within human- and mouse-derived cell lines and in mice. This research has shown that the effectors have at least three broad physiological functions: (1) inhibition of innate immune signalling pathways, (2) suppression of T cell responses and (3) bacterial survival and replication within host cells [10]. This latter function involves the effectors SseF, SseG, SifA, PipB2 and SteA, whose interlinked activities define the unique characteristics of the SCV [10]. While as much as half of the *S*. Typhimurium effector gene repertoire is absent among sequenced strains of *S*. Typhi (either as complete gene absence or presence as pseudogenes [28, 29], it is noteworthy that genes encoding SseF, SseG, SifA, PipB2 and SteA all appear intact, conserved and functional within most sequenced strains of *S*. Typhi [29, 30]. While there has been little work on these ‘core’ effectors in this serovar, there is an extensive literature on their *S*. Typhimurium counterparts, so this knowledge can be extrapolated to help understand the contribution of the *S*. Typhi injectisome to its intracellular growth.

In *S*. Typhimurium, *sseF*, *sseG*, *steA* and *sifA* single mutants are all attenuated for growth in mouse macrophages [7, 12, 31–33]. SseF and SseG are integral membrane proteins that heterodimerize in the SCV membrane and enable efficient bacterial replication in both epithelial cells and macrophages [34–36]. In epithelial cells, they form a tethering complex with the Golgi network-associated protein ACBD3 and contribute to recruitment of membrane to SCVs from a *trans*-Golgi network-derived pathway. This could provide membrane for expanding SCVs and/or nutrients for bacterial growth [37]. Translocated SifA is anchored in the SCV membrane [38]. It prevents the SCV from maturing into a bactericidal phagolysosome [39] and is required for formation of tubules that emanate from SCVs containing either *S*. Typhimurium or *S*. Typhi and have been implicated in intracellular *S*. Typhimurium nutrient acquisition [40]. In macrophages, the SCV membrane also protects bacteria from cytosolic sensors that trigger macrophage cell death and other innate immune responses [41–44]. Without SifA, the membrane surrounding *S*. Typhimurium is unstable [32] and its rupture requires the activity of other effectors including SseJ [45] and SopD2 [46]. SteA is a phospholipid-binding protein that also localizes to the SCV and SCV-derived tubules [47]. PipB2 also localizes to the SCV membrane, where it interacts with a microtubule motor to help drive SCV membrane tubulation [48, 49]. Collectively, these studies indicate that the core effectors cooperate to regulate SCV membrane composition and dynamics, which in turn facilitates intracellular replication of *Salmonella *.

Reuter *et al*. found only low levels of cytosolic *sifA* mutant *S*. Typhi, concluding that this might reflect lower levels of proliferation and lower dependence on SifA function to maintain the integrity of the expanding SCV [13]. However, *sseJ* and *sopD2* are frequently pseudogenes in *S*. Typhi [10] and if non-functional in the *S*. Typhi strain used by Reuter *et al*. [13], an alternative conclusion is that it is this that explains this stability of the SCV membrane enclosing *sifA* mutant *S*. Typhi. Regardless, Hamblin *et al*. [15] detected a replication defect of *S*. Typhi *pipB2* and *sifA* mutants at 24 h post-uptake, indicating that the encoded proteins contribute to replication of *S*. Typhi in human THP-1 macrophages.

With respect to other SPI-2 injectisome effectors in *S*. Typhi, most strains contain intact genes for *pipB*, *sseL* and *sifB*, whose functions are poorly understood. Their apparent functionality suggests that they are under selection and carry out important physiological activities. Clearly, further study of these effectors is needed to improve our understanding of the intracellular biology of *S *. enterica in general. Strikingly, most *S*. Typhi strains lack several effectors (including SseK1, SseK2, SseK3, SspH1, SpvC, SpvD, GtgA and GogB) that in *S*. Typhimurium interfere with innate immune signalling pathways [10]. It is possible that their absence relates to the presence in *S*. Typhi of the polysaccharide Vi capsule, which is linked to a lack of intestinal inflammation by interfering with access of pathogen-associated molecular patterns (PAMPs) to pattern recognition molecules, resulting in less proinflammatory cytokine production [50, 51]. Sequenced genomes of *S*. Typhi contain an intact copy of a gene encoding the SPI-2 injectisome effector SteD [10]; in *S*. Typhimurium SteD suppresses the activation of T cell responses [52, 53], which are known to be crucial for the elimination of *Salmonella* in humans [54, 55].

It remains possible that the main host cell type for *S*. Typhi SPI-2 effector function is epithelial cells [13], but since human macrophages are likely to be an important niche for intracellular growth of *S*. Typhi in deeper tissues [54], it seems unlikely that the functions of the core effectors discussed above would have no or little effect on growth in this environment. Based on the evidence discussed above, it seems premature to discount a role for the *S*. Typhi SPI-2 injectisome in intramacrophage growth, and we suggest that more studies need to be done, involving a detailed analysis of the secretion kinetics of individual effectors of the two serovars, more extended periods of infection than those used in the majority of experiments to date and using more sensitive assays for bacterial replication than bulk c.f.u. counts.
